# Emotional resilience and its role in promoting well-being and employability during the school-to-work transition under labor market uncertainty

**DOI:** 10.3389/fpsyg.2026.1783091

**Published:** 2026-03-11

**Authors:** Shengying Yang, Qixiu Qin, Yunxuan Wang

**Affiliations:** 1 School of Computer Science and Technology, Zhejiang University of Science and Technology, Hangzhou, Zhejiang, China; 2 School of Foreign Languages, China West Normal University, Nanchong, China; 3 School of International Education, St Luke’s Campus, University of Exeter, Exeter, United Kingdom

**Keywords:** emotional resilience, employability, labor market uncertainty, psychological resources and career development, school-to-work transition, well-being

## Abstract

The transition from higher education into employment has become more demanding for graduates as economic conditions increase uncertainty surrounding early career opportunities. To examine how psychological resources operate under such conditions, this study empirically tests the relationships among emotional resilience, well-being, labor market uncertainty, and employability using established multidimensional measures. The results indicate that emotional resilience is positively associated with employability, both through a direct pathway and through an indirect pathway operating via well-being. Specifically, higher levels of emotional resilience correspond to stronger well-being, which in turn relates to greater career adaptability, motivation, and confidence. The analysis further shows that labor market uncertainty conditions these relationships. When perceived uncertainty is higher, the positive association between emotional resilience and well-being is reduced, and the link between emotional resilience and employability is correspondingly weakened. This pattern suggests that the effectiveness of psychological resources is contingent on the stability of the external employment environment. Additional analyses using alternative model specifications yield consistent results, supporting the reliability of the observed relationships. Taken together, the findings indicate that employability is not solely determined by individual psychological strengths, but emerges from their interaction with labor market conditions. This underscores the importance of institutional efforts that simultaneously foster students’ emotional resilience and well-being while reducing informational ambiguity during the school-to-work transition through clearer guidance and structured support mechanisms.

## 1 Introduction

### 1.1 School-to-work transitions under labor market uncertainty

In many national contexts, economic shocks, industrial restructuring, and technological transformation have reduced the predictability of employment conditions for young labor market entrants, making the transition from education to work increasingly uncertain. Graduates frequently encounter ambiguity regarding job availability and career prospects, and longitudinal evidence shows that periods of economic contraction are associated with lower well-being among job seekers entering the labor market (Morgan and O’Connor, 2022). Changes in job design and employment arrangements have further reshaped early career experiences, as evolving role expectations and blurred work boundaries are linked to declines in well-being even in flexible work settings (Hennecke and Knabe, 2025). Similar patterns are observed in rapidly transforming economies, where graduates often face skill mismatches and limited job opportunities that increase insecurity during the transition from education to employment (Isirabahenda, 2025). Institutional contexts also influence how uncertainty is experienced, with weakly implemented labor market policies contributing to stronger perceptions of insecurity and pressure among job seekers (Sage, 2015). Expanding temporary and informal employment arrangements are likewise associated with lower perceived job stability and reduced well-being among young workers (Karabchuk and Soboleva, 2020). Over time, structural differences in educational pathways may further widen disparities in career trajectories, reinforcing uncertainty perceptions during early employment stages (Decker et al., 2025). Rather than representing only an objective labor market condition, such uncertainty is increasingly understood as a psychologically perceived context that shapes individuals’ emotional responses and career decision-making processes during labor market entry. When employment prospects appear unstable or unclear, individuals report elevated stress and reduced confidence in career planning, which may hinder successful adjustment during early career development. For university students approaching graduation, these conditions intensify the psychological demands of the school-to-work transition and increase reliance on personal psychological resources to cope with employment-related challenges.

Early research on emotional experience and psychological adaptation provides the conceptual basis for understanding emotional resilience and its relationship with psychological well-being. Cognitive and emotion-regulation processes shape how individuals respond to life events and maintain psychological functioning, thereby influencing subjective well-being (Şimşek, 2011). Subsequent work examining personality-related emotional functioning demonstrates that emotional stability and extraversion are positively associated with well-being, situating resilience-related differences within broader adjustment processes rather than clinical pathology (Morris et al., 2015). Within educational research, emotional competencies are increasingly conceptualized as developable capacities rather than fixed traits, and structured positive psychology interventions have been shown to enhance emotion regulation, resilience, and learning-related motivation among students (Farrer, 2013). Recent studies extend these perspectives to career development contexts, suggesting that psychological resources play a central role in preparing students for labor market entry, as learning experiences combined with emotional health support career readiness beyond skill acquisition alone (Verma and Anand, 2025). Empirical evidence further indicates that students’ emotional states and academic engagement are associated with employability-related competencies, with career adaptability acting as an explanatory mechanism (Jiang et al., 2024). Longitudinal findings provide additional support by demonstrating bidirectional associations between psychological well-being and self-perceived employability over time, such that higher well-being contributes to clearer vocational direction while stronger employability perceptions reinforce psychological stability (Petruzziello et al., 2025). Collectively, these studies suggest that emotional resilience contributes to employability partly through its association with psychological well-being during the transition from university to the labor market.

Despite growing recognition of the importance of emotional resources in career development, important questions remain unresolved regarding how these psychological processes operate under conditions of labor market uncertainty. Prior research indicates that the relationship between career resilience and well-being may vary depending on experiences of achievement and stress during employment entry, suggesting that resilience does not function uniformly across transition stages (Han et al., 2021). Related evidence shows that emotional resilience is associated with improved well-being and employment-related competencies, although these effects differ across learning environments and job-search contexts (Feng and Wang, 2025). University experiences have also been shown to shape students’ perceptions of employability, yet the mechanisms through which emotional processes translate educational experiences into employability evaluations remain insufficiently specified (Schettino et al., 2022). From a career psychology perspective, negative career thoughts may weaken adaptability during early career development, indicating that emotional processes influence adjustment but leaving unclear how these effects extend into active job-search behavior (Faria, 2021). Similarly, research on graduate populations identifies links between emotional intelligence and well-being without examining how these psychological attributes subsequently influence employability judgments at labor market entry (Larguinho et al., 2025). Existing research therefore leaves three issues insufficiently addressed: how emotional resilience functions under perceived labor market uncertainty, whether well-being serves as a mechanism linking resilience to employability, and how these relationships unfold during the school-to-work transition. To address these gaps, the present study proposes a moderated mediation framework in which emotional resilience influences employability both directly and indirectly through well-being, while labor market uncertainty conditions the strength of these relationships.

## 2 Review

### 2.1 Emotional resilience as a psychological resource in the university-to-work transition

Emotional resilience refers to individuals’ capacity to regulate emotions, recover from stress, and maintain psychological functioning when facing demands and uncertainty. This capacity is particularly relevant during the transition from university to the labor market, a period characterized by academic pressure, career decision-making, and anticipated employment uncertainty. Research consistently indicates that emotional resilience is closely associated with psychological well-being among students, as individuals with stronger emotion-regulation and recovery abilities maintain more stable well-being under academic and interpersonal stress (Suh, 2016). Evidence from intervention-based studies further suggests that emotional resilience is not a fixed trait but a developable capacity, with structured emotion-regulation training leading to improved resilience and more stable stress responses (Hemati et al., 2020). Related research highlights the broader psychological functions of resilience in educational settings. Students with higher levels of resilience demonstrate stronger links between emotional competence and psychological well-being as well as improved academic performance, indicating that resilience supports adaptive functioning across multiple psychological domains (George William, 2022). Similar patterns have been observed beyond student samples, where higher levels of self-compassion and mindfulness are associated with stronger emotional resilience and more adaptive emotional functioning under stress (Sabir et al., 2018). Studies focusing specifically on higher education contexts likewise show that emotional resilience contributes to subjective well-being by facilitating faster recovery from setbacks and more effective adaptation to academic challenges (Safaee-Rad, 2015). Research has also connected emotional resilience to decision-related processes relevant to early career development. Resilience, personality dispositions, and decision-making competence jointly contribute to emotional stability and judgment quality when graduates confront uncertain labor market conditions (Venkatesan and Rohatgi, 2018). These findings suggest that emotional resilience supports psychological adjustment by enabling individuals to manage uncertainty and maintain positive functioning during periods of transition. Accordingly, emotional resilience can be understood as a foundational psychological resource that promotes well-being during the school-to-work transition, providing a theoretical basis for examining its indirect influence on employability through psychological well-being.

### 2.2 Well-being as a mediating mechanism in transitional adaptation

During the transition from university to employment, students face academic demands, career-related decision-making, and uncertainty regarding future work roles, making psychological well-being an important factor in successful adjustment to labor market entry. Psychological well-being is increasingly understood as a resource that supports coping processes, sustained engagement, and adaptive functioning rather than merely reflecting affective experience. Empirical research shows that emotional intelligence contributes to higher subjective well-being through adaptive emotional expression and humor use, which in turn facilitates more effective stress management among university students (Wang et al., 2019). Related findings indicate that emotional intelligence and mindfulness jointly support stable well-being, and such psychological stability is associated with greater decisional clarity when young adults confront uncertain career expectations (Liu et al., 2021). Well-being is also closely connected to motivational and behavioral processes relevant to early career development. Positive affective states have been shown to enhance engagement and career satisfaction in work contexts, patterns that similarly emerge during the early stages of labor market entry (Joo and Lee, 2017). Research on emotional labor further demonstrates that difficulties in managing emotional demands are associated with reduced motivation and poorer adjustment when individuals encounter unfamiliar work tasks, suggesting that well-being plays a role in facilitating adaptive responses to new occupational environments (Cheung et al., 2018). Additional psychological resources contribute to this process, as higher levels of spiritual intelligence are linked to stronger emotional well-being and greater psychological stability under conditions of uncertainty (Jamwal and Kamboj, 2025). Beyond individual psychological traits, broader economic and institutional conditions are also associated with fluctuations in well-being. Evidence indicates that labor market conditions influence individuals’ emotional evaluations and sense of purpose, highlighting the role of well-being in shaping how people interpret and respond to career opportunities (Tay and Harter, 2013). Taken together, these findings suggest that psychological well-being supports motivation, decision-making clarity, and adaptive engagement during career entry, thereby contributing to individuals’ readiness for employment. Accordingly, well-being can be conceptualized as a key psychological mechanism through which emotional resources are translated into employability outcomes during the school-to-work transition.

### 2.3 Labor market uncertainty as a contextual boundary condition

Labor market uncertainty represents an important contextual condition influencing young people’s psychological adjustment and career development during the transition from education to employment. Rather than operating solely as a structural characteristic of economic systems, labor market uncertainty shapes individuals’ perceptions of career opportunities and influences how psychological resources are mobilized during labor market entry. Empirical evidence indicates that instability in the external employment environment is associated with lower career confidence and slower adjustment among graduates entering the workforce (Mucea, 2022). Research further shows that individuals engaged in less stable forms of labor participation report lower levels of well-being and stronger stress responses, suggesting that perceived employment insecurity carries significant psychological costs (Qu, 2022). Importantly, uncertainty is often experienced through concrete frictions at the education–work interface, such as employability skills mismatch and employers’ shifting demands, which can intensify graduates’ sense of unpredictability and weaken confidence in career planning (Kalei, 2015). In addition, labor market returns are not uniform across fields of study or demographic groups; evidence from graduate labor market performance suggests that specialization patterns and language/major choices can yield uneven employment outcomes, thereby amplifying perceived uncertainty for certain groups even within the same macroeconomic context (Kim et al., 2014). Job characteristics and employment conditions also contribute to emotional strain during early career development. Unequal access to employment opportunities and discriminatory workplace environments are associated with reduced emotional health and poorer employment quality, particularly among individuals facing structural disadvantages (Drydakis, 2017). Socio-cultural contexts further shape how individuals evaluate their well-being, as external pressures influence subjective assessments of psychological functioning across cultural settings (Uotinen et al., 2025). Moreover, graduate transitions in the post-pandemic period show that disrupted early-career pathways and volatile hiring climates can shape educational outcomes and employability perceptions, reinforcing the idea that uncertainty is not only macro-level but also embedded in cohort-specific transition conditions (Aribe and Rojo, 2025). Among young people, labor market uncertainty is closely linked to career motivation and decision-making processes, with higher stress levels predicting lower perceived employability, reduced career confidence, and increased emotional exhaustion during labor market entry (Nimmi and Donald, 2023). Even among postgraduate populations, employability and career alignment are intertwined with expectations about sustainable futures and the perceived clarity of occupational trajectories, indicating that uncertainty can persist beyond initial graduation and influence how individuals appraise their long-term career fit (Embornas and Labadisos, 2024). Similarly, evidence from tracer studies suggests that advanced education does not automatically translate into predictable career progression, and uncertainty about returns to graduate education may shape how individuals evaluate employability and plan subsequent career steps (Maghamil, 2025). Although emotional intelligence and coping strategies can mitigate stress responses, negative associations between unstable labor conditions and psychological health remain evident, indicating that contextual uncertainty constrains individual adaptation processes (Eranhikkal and Ahirwar, 2025). Longitudinal evidence further demonstrates that changes in labor market participation are associated with fluctuations in emotional well-being, with these associations becoming stronger under less stable macroeconomic conditions (Lewin and Stier, 2025). Collectively, these findings suggest that labor market uncertainty does not merely influence career outcomes directly but alters the psychological conditions under which individuals regulate emotions and maintain well-being during career transitions. Accordingly, labor market uncertainty can be conceptualized as a contextual moderator that conditions the strength of relationships between emotional resilience, psychological well-being, and employability during the school-to-work transition.

## 3 Methods

### 3.1 Research design and analytical framework

This study employs structural equation modeling (SEM) to examine the relationships among emotional resilience, well-being, labor market uncertainty, and employability during students’ transition from higher education to employment. The analytical framework integrates perspectives from resilience research, subjective well-being theory, vocational development, and labor market psychology to explain how psychological resources operate under conditions of perceived employment uncertainty. Data were collected through a structured questionnaire administered to university students approaching graduation in China. The survey was distributed via Wenjuanxing, a widely used online survey platform in Chinese academic research that enables anonymous participation and prevents duplicate submissions through device and IP restrictions. The final analytic sample consisted of 400 valid responses, obtained after data screening procedures that removed incomplete submissions and invalid response patterns. Participants were recruited through university-based distribution channels, and participation was voluntary and anonymous.

All study variables were measured using multi-item scales adapted from established instruments in resilience, well-being, and employability research. Responses were recorded using a five-point Likert scale ranging from 1 (strongly disagree) to 5 (strongly agree). Composite scores were computed by averaging item responses within each construct. The model conceptualizes emotional resilience as an individual psychological resource reflecting students’ capacity to regulate emotions, recover from stress, and maintain functioning under transition-related demands. Well-being is specified as a mediating variable representing psychological stability and positive self-evaluation during adjustment to career transition. Employability is operationalized as a multidimensional perception encompassing career preparedness, confidence in transferable skills, and engagement with employment opportunities. Labor market uncertainty is modeled as a contextual moderator capturing perceived instability and ambiguity in employment prospects. All analyses were conducted using Python (version 3.13) within an Anaconda environment on macOS. Structural equation modeling and confirmatory factor analyses were estimated using the semopy package, while data processing and statistical diagnostics were implemented using pandas, numpy, scipy, and statsmodels. Reliability and validity indices (Cronbach’sα, composite reliability, and AVE) were computed programmatically, and moderation and robustness analyses were performed using ordinary least squares regression with heteroskedasticity-consistent standard errors and bootstrap resampling procedures (5,000 iterations). Visualization and simple-slope analyses were generated using matplotlib and seaborn. The SEM framework simultaneously estimates direct effects, indirect (mediated) effects, and moderated relationships within a unified model. This integrated approach enables examination of how individual psychological characteristics and perceived labor market conditions jointly relate to employability outcomes during the university-to-work transition, while also allowing robustness checks across alternative estimation strategies to ensure the stability of parameter estimates.

### 3.2 Measurement framework

**Figure 1:**
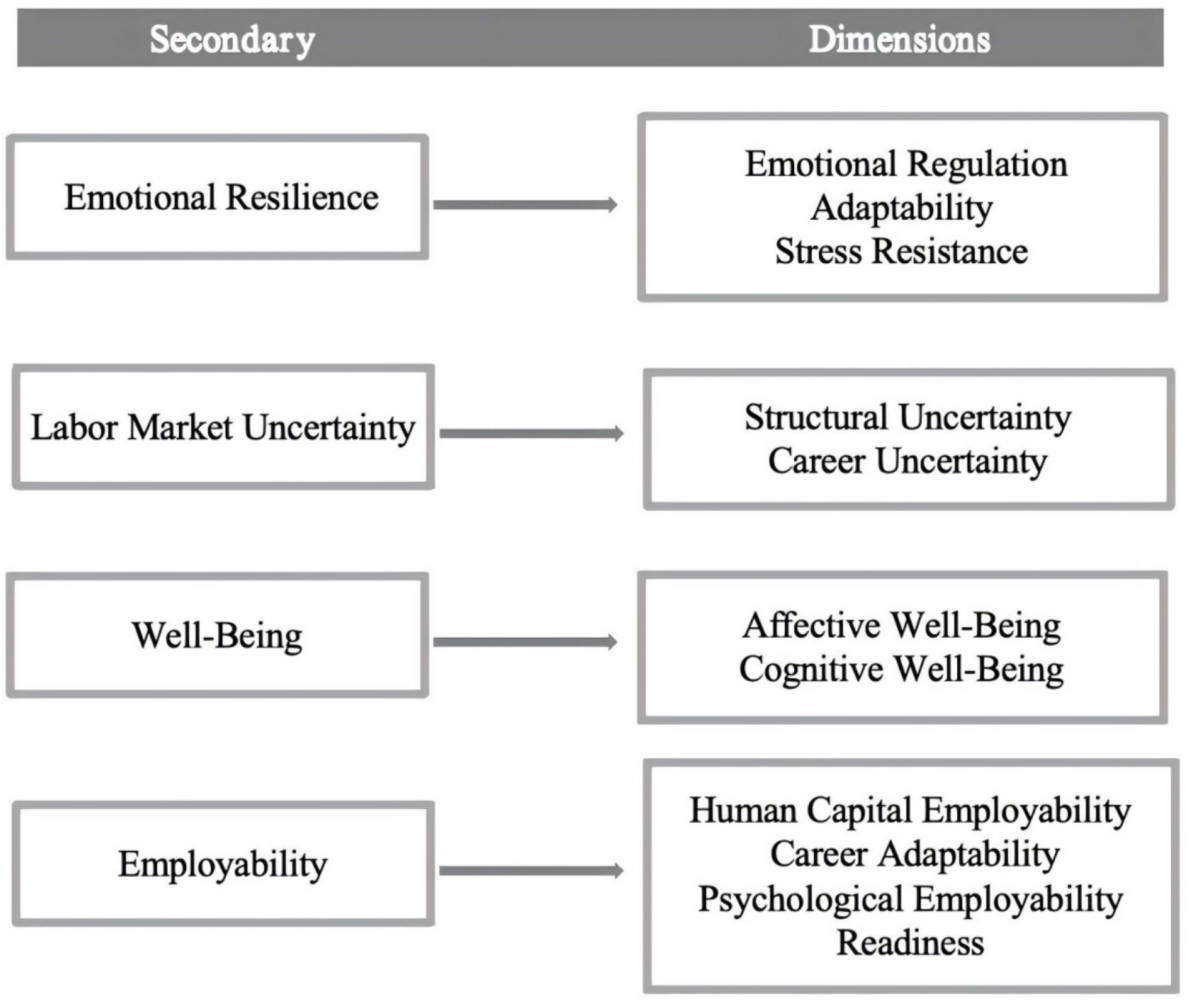
Measurement framework of core constructs and their dimensions.

To operationalize the study variables, this study specifies a measurement structure for the core constructs. Figure 1 summarizes the measurement structure used in this study, which includes four constructs: emotional resilience, labor market uncertainty, well-being, and employability. Each construct is measured using multiple dimensions derived from established scales, ensuring that the theoretical concepts are represented through observable indicators. The structure shown in Figure 1 covers key psychological resources, perceptions of the labor market environment, and employability related outcomes that are relevant to students during the transition from university to the labor market. By specifying the dimensions associated with each construct, the figure defines how the variables are measured and distinguished at the empirical level. This measurement specification is applied in the subsequent analyses to test direct and indirect relationships among the core constructs, as well as moderation effects associated with labor market uncertainty.

## 4 Results

### 4.1 Assessment of measurement reliability, validity, and model fit

Table 1 indicates that the measurement model satisfies commonly accepted psychometric standards. All standardized factor loadings exceed the recommended threshold of 0.70, ranging from 0.71 to 0.92, suggesting that the observed indicators adequately represent their respective latent constructs. Internal consistency is satisfactory across all scales, with Cronbach’s alpha values ranging from 0.85 to 0.87, while composite reliability (CR) values fall between 0.89 and 0.90, exceeding conventional criteria and indicating stable measurement reliability. Convergent validity is also supported, as the average variance extracted (AVE) values range from 0.68 to 0.70, all above the recommended cutoff of 0.50, demonstrating that a substantial proportion of variance in the observed indicators is explained by their underlying constructs. Compared with the other variables, labor market uncertainty exhibits a slightly wider range of factor loadings (0.71–0.92), suggesting greater heterogeneity among its indicators, which is consistent with the multidimensional and perception-based nature of uncertainty constructs; however, its CR and AVE values remain within acceptable ranges, confirming adequate measurement quality. Overall, these results indicate that all four latent constructs demonstrate satisfactory reliability and convergent validity, providing a robust measurement foundation for subsequent structural analyses examining the relationships among emotional resilience, well-being, labor market uncertainty, and employability during the university-to-work transition.

**Table 1:** Measurement model reliability, validity, and fit indices.

Construct	Factor loadings	Cronbach’s α	CR	AVE
Emotional resilience (ER)	0.72–0.87	0.85	0.89	0.68
Labor market uncertainty (LMU)	0.71–0.92	0.85	0.89	0.68
Well-being (WB)	0.74–0.89	0.86	0.9	0.68
Employability (EMP)	0.76–0.90	0.87	0.9	0.7

### 4.2 Descriptive statistics, correlation structure, and multicollinearity diagnostics

Table 2 reports the descriptive statistics, correlations, and multicollinearity diagnostics among emotional resilience, labor market uncertainty, well-being, and employability. Emotional resilience is positively correlated with both well-being (*r* = 0.55, *p* < 0.001) and employability (*r* = 0.55, *p* < 0.001), indicating that students with higher levels of resilience tend to report better psychological well-being and stronger perceptions of employability during the transition from university to employment. Emotional resilience is negatively correlated with labor market uncertainty (*r* = −0.14, *p* < 0.01), suggesting that individuals perceiving greater employment uncertainty tend to report lower levels of resilience. Labor market uncertainty also shows significant negative correlations with well-being (*r* = −0.39, *p* < 0.001) and employability (*r* = −0.38, *p* < 0.001), indicating that higher perceived uncertainty is associated with poorer psychological adjustment and weaker employability perceptions. In contrast, well-being is strongly and positively correlated with employability (*r* = 0.63, *p* < 0.001), reflecting a close association between students’ psychological functioning and their perceived readiness for career entry. Variance inflation factor (VIF) values range from 1.25 to 1.97, all well below commonly accepted thresholds, indicating that multicollinearity is not a concern among the study variables. These correlation patterns provide preliminary empirical support for the hypothesized relationships and justify further examination of the direct, indirect, and moderated effects tested in the structural model.

**Table 2:** Descriptive statistics, correlations, and multicollinearity diagnostics.

Variable	M	SD	1	2	3	4	VIF
1. ER	2.98	0.84	—				1.63
2. LMU	3.01	0.84	−0.14**	—			1.25
3. WB	2.99	0.85	0.55***	−0.39***	—		1.97
4. EMP	2.99	0.85	0.55***	−0.38***	0.63***	—	1.93

***p < 0.001; **p < 0.01; *p < 0.05.

### 4.3 Tructural path estimates for emotional resilience, labor market uncertainty, well-being, and employability

**Figure 2:**
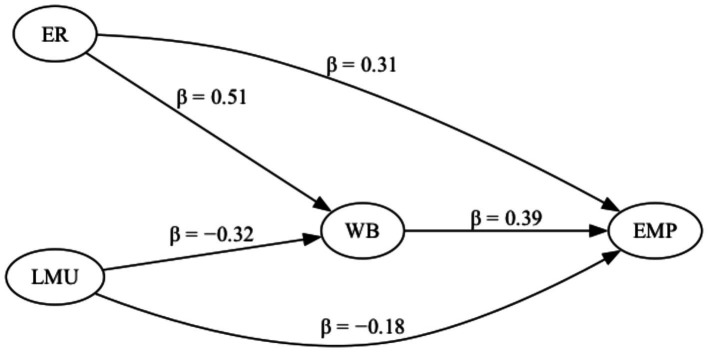
Structural path diagram with standardized coefficients (*β*) for the effects of emotional resilience (ER) and labor market uncertainty (LMU) on well-being (WB) and employability (EMP).

Table 3 and Figure 2 present the estimated structural paths among emotional resilience, well-being, labor market uncertainty, and employability. Emotional resilience shows a significant positive association with well-being (*β* = 0.51, *t* = 12.90, *p* < 0.001), indicating that higher levels of resilience are related to higher psychological well-being during the transition from university to employment. Well-being, in turn, is positively associated with employability (*β* = 0.39, *t* = 8.41, *p* < 0.001), suggesting that students reporting greater well-being also perceive stronger employability. In addition to this indirect pathway, emotional resilience maintains a significant direct association with employability (*β* = 0.31, *t* = 7.09, *p* < 0.001), indicating that resilience contributes to employability beyond its relationship with well-being. Labor market uncertainty is negatively associated with both well-being (*β* = −0.32, *t* = −8.15, *p* < 0.001) and employability (*β* = −0.18, *t* = −4.65, *p* < 0.001), suggesting that higher perceived uncertainty corresponds to lower psychological well-being and weaker employability perceptions. Figure 2 illustrates the pattern of positive pathways linking emotional resilience to well-being and employability alongside the negative pathways associated with labor market uncertainty, indicating that employability during the school-to-work transition is jointly related to individual psychological resources and perceived labor market conditions.

**Table 3:** Structural path coefficients among key constructs.

Path	*β*	SE	t	*p*	Sign.
ER → WB	0.51	0.04	12.9	< 0.001	***
LMU → WB	−0.32	0.04	−8.15	< 0.001	***
ER → EMP	0.31	0.04	7.09	< 0.001	***
WB → EMP	0.39	0.05	8.41	< 0.001	***
LMU → EMP	−0.18	0.04	−4.65	< 0.001	***

***p < 0.001; **p < 0.01; *p < 0.05.

### 4.4 Indirect effects of emotional resilience on employability through well-being

**Figure 3:**
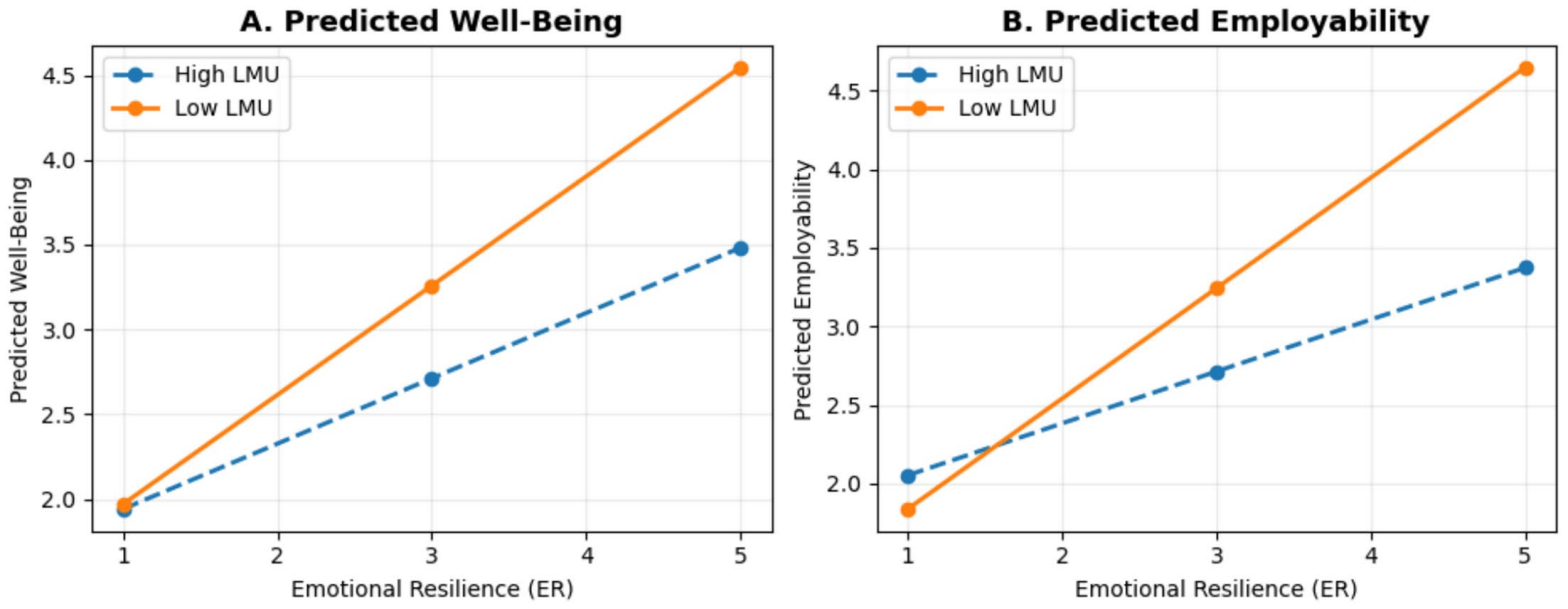
Simple slopes of the moderating effect of labor market uncertainty on the relationships between emotional resilience and outcomes. **(A)** Predicted well-being. **(B)** Predicted employability.

Table 4 reports a significant indirect effect of emotional resilience on employability through well-being (*β* = 0.26, 95% CI [0.20, 0.33], *p* < 0.001), supporting the hypothesized mediating role of well-being in the relationship between emotional resilience and employability. The result indicates that emotional resilience is associated with employability partly via its positive association with students’ well-being, rather than operating only through a direct pathway. Specifically, higher emotional resilience is related to higher well-being, which is in turn related to stronger self-assessed employability, suggesting that well-being accounts for a meaningful portion of how resilience translates into employability perceptions during the university-to-work transition. This mediation pattern implies that students’ psychological states contribute to the extent to which resilience is reflected in perceived career readiness and confidence in managing early career demands under uncertain employment conditions (Figure 3).

**Table 4:** Mediation effects of well-being (bootstrap estimates).

Mediation path	Indirect effect	95% CI	*p*	Sign.
ER → WB → EMP	0.26	[0.20, 0.33]	< 0.001	***

***p < 0.001; **p < 0.01; *p < 0.05.

### 4.5 Moderating role of labor market uncertainty in psychological and career-related pathways

Table 5 reports the moderation results testing whether labor market uncertainty changes the strength of the relationships between emotional resilience and (a) well-being and (b) employability. The interaction term predicting well-being is negative and significant (ER × LMU → WB: *β* = −0.11, SE = 0.03, *t* = −3.48, *p* < 0.001), indicating that the positive association between emotional resilience and well-being becomes weaker as perceived labor market uncertainty increases. The interaction term predicting employability is also negative and significant (ER × LMU → EMP: *β* = −0.16, SE = 0.03, *t* = −5.05, *p* < 0.001), showing that higher uncertainty similarly attenuates the positive association between emotional resilience and employability. In substantive terms, these results support a context-contingent interpretation of psychological resources: emotional resilience is more strongly linked to favorable psychological and career-related outcomes when students perceive the labor market as relatively stable, whereas under higher perceived uncertainty the benefits of resilience for well-being and employability are reduced, consistent with the simple-slope pattern reported in Table 6.

**Table 5:** Moderation effect estimates (interaction terms).

Interaction path	*β*	SE	t	*p*	Sign.
ER × LMU → WB	−0.11	0.03	−3.48	< 0.001	***
ER × LMU → EMP	−0.16	0.03	−5.05	< 0.001	***

***p < 0.001; **p < 0.01; *p < 0.05.

**Table 6:** Simple slope estimates under high vs. low labor market uncertainty.

Condition	Slope (ER → EMP)	*p*	Sign.
Low LMU (−1 SD)	0.59	< 0.001	***
High LMU (+1 SD)	0.28	< 0.001	***

***p < 0.001; **p < 0.01; *p < 0.05.

Table 6 reports the simple slope estimates for the association between emotional resilience and employability at different levels of labor market uncertainty. The results show that this association differs across uncertainty conditions. Under low perceived labor market uncertainty (−1 SD), emotional resilience is more strongly associated with employability (*β* = 0.59, *p* < 0.001), indicating a robust positive relationship between resilience and perceived employability. Under high perceived labor market uncertainty (+1 SD), the slope remains positive but is weaker (*β* = 0.28, *p* < 0.001), indicating that higher uncertainty attenuates the strength of the resilience–employability association. The contrast between these slopes suggests that the contribution of emotional resilience to employability is more pronounced when students perceive the labor market as relatively stable and less pronounced when uncertainty is higher, providing evidence that labor market uncertainty conditions how emotional resilience relates to employability during the university-to-work transition.

### 4.6 Robustness and model validation analyses

Table 7 presents robustness and model validation analyses examining whether the structural relationships identified in the hypothesized model remain stable across alternative analytical specifications. The model integrating emotional resilience, well-being, labor market uncertainty, and employability demonstrates satisfactory performance and consistent parameter estimates across estimation approaches. Bootstrap analyses based on 5,000 resamples produce coefficient estimates highly comparable to those obtained from the primary structural equation model, indicating that the observed relationships are not driven by sampling variability. Additional robustness checks further show that the key structural paths remain statistically significant when alternative estimation strategies are applied, suggesting that the relationships among emotional resilience, well-being, labor market uncertainty, and employability are stable and not sensitive to methodological variation. Across specifications, emotional resilience maintains positive associations with both well-being and employability, whereas labor market uncertainty consistently shows negative associations with these outcomes, with effect magnitudes remaining comparable in direction and size. This pattern indicates that employability outcomes are better explained when psychological resources and perceived labor market conditions are considered jointly rather than independently. The stability of parameter estimates across robustness analyses further supports the interpretation of well-being as a mediating mechanism linking emotional resilience to employability and of labor market uncertainty as a contextual moderator shaping the strength of psychological effects during the university-to-work transition. The convergence of findings across analytical approaches therefore reinforces confidence that the hypothesized model provides a reliable representation of the relationships among the study variables and that emotional resilience, psychological well-being, and perceived labor market uncertainty jointly contribute to explaining variation in employability during the school-to-work transition.

**Table 7:** Robustness and model validation analyses.

Robustness test	ER → WB	LMU → WB	WB → EMP	ER → EMP	LMU → EMP	Conclusion
Main model (standardized path estimates)	0.50***	−0.32***	0.39***	0.31***	−0.18***	Supported
Bootstrap (5,000 resamples; beta mean [95% CI])	0.50***	−0.32 ***	0.39 ***	0.31 ***	−0.18 ***	Stable
Robust SE (HC3) significance check	0.50***	−0.32***	0.39***	0.31***	−0.18***	Stable
OLS check (standardized variables)	0.50***	−0.32***	0.39***	0.31***	−0.18***	Consistent
Raw-score check (unstandardized coefficients)	0.511***	−0.320***	0.391***	0.311***	−0.183***	Direction unchanged

***p < 0.001; **p < 0.01; *p < 0.05.

## 5 Discussion

### 5.1 Psychological resources as stabilizing forces in an uncertain labor market

The structural relationships identified in this study indicate that emotional resilience is positively associated with both well-being and employability during the transition from university to the labor market, suggesting that students with higher levels of emotional resilience are more likely to maintain positive psychological functioning and stronger perceptions of employability under conditions characterized by labor market uncertainty. This finding is consistent with prior research demonstrating that emotional resilience supports psychological adjustment and emotional stability when students face academic and career-related stressors (Suh, 2016), while extending this line of work by showing that resilience remains relevant for employability perceptions during early career transitions. At the same time, labor market uncertainty is negatively associated with both well-being and employability, indicating that perceived instability in the employment environment corresponds to more cautious emotional and career-related evaluations among students, a pattern aligned with previous findings linking unstable employment environments to reduced psychological well-being and lower career confidence among young labor market entrants (Mucea, 2022). Taken together, these results suggest that students’ career adjustment reflects the joint influence of internal psychological resources and perceptions of external opportunity structures rather than either factor operating independently. The findings further indicate that emotional resilience remains positively related to well-being and employability even under conditions of uncertainty, while perceived labor market instability exerts concurrent negative associations with these outcomes, highlighting that psychological resources operate within contextual constraints rather than independently of environmental conditions. Students may therefore experience psychological preparedness for career entry while simultaneously perceiving limitations in available opportunities. Overall, the results support an interpretation of early career development in which psychological resources and perceived labor market conditions jointly shape students’ experiences during the school-to-work transition, with emotional resilience associated with sustained engagement in career-related processes and perceived uncertainty contributing to more cautious evaluations of employability, thereby helping explain variation in early career trajectories even among students with comparable internal psychological resources.

### 5.2 The translational role of well-being in converting emotional resilience into employability

The mediation analysis indicates that well-being accounts for a substantial portion of the association between emotional resilience and employability, demonstrating that emotional resilience relates to employability both directly and indirectly through students’ psychological well-being. Consistent with the structural model results, emotional resilience shows a significant direct association with employability while also exerting an indirect effect via well-being, suggesting that resilience contributes to employability partly through its positive relationship with students’ psychological functioning during the transition from university to employment. This finding aligns with prior research linking emotional functioning and well-being to employability-related outcomes among university students (Jiang et al., 2024) and extends existing work by empirically identifying well-being as a mediating mechanism within the school-to-work transition context. Specifically, higher emotional resilience is associated with greater well-being, which in turn corresponds to more favorable evaluations of employability, indicating that well-being captures an important psychological process through which resilience is translated into perceptions of career readiness. Rather than functioning solely as an emotional outcome, well-being appears to represent the psychological conditions under which personal resources are effectively expressed in career-related judgments and self-evaluations. This interpretation is consistent with perspectives on psychological capital suggesting that resilience provides the capacity to manage demands, whereas well-being reflects the experiential state enabling this capacity to influence behavioral and evaluative outcomes. The results therefore highlight the distinct yet complementary roles of emotional resilience and well-being in shaping students’ career development during the school-to-work transition. Importantly, the mediating role of well-being remains meaningful under conditions of labor market uncertainty, where students’ emotional experiences fluctuate alongside their assessments of career opportunities, suggesting that employability is associated not only with psychological strengths themselves but also with the level of well-being through which these strengths are translated into career-related evaluations. From a practical perspective, these findings imply that initiatives aimed at enhancing students’ employability may benefit from simultaneously supporting psychological well-being and strengthening emotional resilience, particularly in employment environments characterized by uncertainty and transition-related stress.

### 5.3 Contextual limits of psychological capital under labor market uncertainty

The moderation analysis indicates that the associations between emotional resilience and both well-being and employability vary systematically across levels of perceived labor market uncertainty, suggesting that the effectiveness of psychological resources depends partly on contextual employment conditions during the school-to-work transition. Specifically, emotional resilience demonstrates a stronger positive association with well-being and employability when perceived labor market uncertainty is low, whereas these relationships become weaker as uncertainty increases. The simple-slope results further illustrate this pattern, showing that although emotional resilience remains positively associated with employability under both conditions, the magnitude of this association is attenuated in contexts characterized by higher perceived instability. These findings are consistent with prior research indicating that external labor market conditions influence psychological adjustment and career-related evaluations during labor market entry (Lewin and Stier, 2025), while extending this literature by demonstrating that uncertainty does not merely relate to outcomes directly but conditions the strength of relationships between internal psychological resources and career-related perceptions. In this sense, labor market uncertainty functions as a contextual boundary condition shaping how emotional resilience is translated into well-being and employability during early career development. The results further suggest that psychological resources do not operate independently of perceived opportunity structures; rather, their associations with adjustment outcomes are embedded within broader environmental perceptions. Although emotional resilience remains positively related to psychological and career-related outcomes, its influence is comparatively reduced when students perceive higher levels of labor market instability, supporting an interactional perspective in which individual capacities and contextual perceptions jointly shape transition experiences. From an applied perspective, these findings imply that interventions focusing solely on strengthening students’ psychological resources may have limited effectiveness if perceptions of labor market uncertainty remain unaddressed. Providing clearer career information, realistic employment expectations, and structured transition support may therefore enhance the extent to which psychological resources translate into favorable employability evaluations. Emotional resilience thus remains an important developmental resource, but its role is conditioned by the broader labor market context faced by students during the transition from university to employment.

## 6 Conclusion

This study examined how emotional resilience, psychological well-being, and perceived labor market uncertainty jointly relate to university students’ employability during the transition from education to work. The findings indicate that emotional resilience is positively associated with employability both directly and indirectly through well-being, highlighting the psychological processes through which individual emotional resources are reflected in perceptions of career readiness. At the same time, perceived labor market uncertainty shows negative associations with well-being and employability and weakens the strength of these relationships, suggesting that psychological resources operate within contextual constraints rather than independently of external employment conditions. By integrating psychological resources and perceived labor market environments within a moderated mediation framework, this study contributes to a more comprehensive understanding of early career development, positioning employability as a dynamic outcome emerging from the interaction between internal psychological capacities and contextual perceptions during the school-to-work transition rather than as a product of individual competencies alone. The results clarify the mechanism through which emotional resilience is translated into employability via well-being while identifying the boundary conditions under which this process becomes stronger or weaker. In doing so, the study advances career development research by emphasizing the joint role of psychological adaptation and perceived labor market environments in shaping students’ transition experiences and provides a foundation for future research examining how evolving labor market contexts influence psychological adjustment and employability outcomes across different institutional and cultural settings.

### 6.1 Limitations and practical implications

The findings of this study should be interpreted with several considerations in mind that also point toward directions for future research and practical application. The cross-sectional nature of the research design limits the extent to which causal relationships among emotional resilience, well-being, labor market uncertainty, and employability can be firmly established. Although the proposed model is theoretically grounded, developmental processes during the school-to-work transition unfold over time, and longitudinal or multi-wave research would provide stronger insight into how psychological resources and perceptions of labor market conditions evolve as students move into early employment stages. The reliance on self-reported measures represents another constraint, as the results reflect subjective perceptions rather than objective labor market conditions. Perceived uncertainty remains theoretically meaningful because career decisions are shaped by individuals’ interpretations of opportunity structures; however, future studies incorporating objective labor market indicators or multi-source data could further clarify how perceived and structural uncertainty interact in shaping career development outcomes. The sample is drawn from university students within a specific educational context, which may limit the generalizability of the findings across institutional and cultural environments. Variations in labor market systems, educational arrangements, and transition support structures may influence the ways in which psychological resources operate during career entry. Comparative and cross-cultural research would therefore contribute to a broader understanding of whether the observed relationships remain stable across different transition contexts.

Despite these constraints, the findings offer several practical implications for higher education institutions and career development policy. The results indicate that employability is shaped not only by individual competencies but also by psychological well-being and perceptions of labor market uncertainty. Integrating psychological resource development into career preparation programs may therefore enhance students’ readiness for labor market entry, particularly through initiatives that strengthen emotional resilience and support well-being during periods of career transition. The moderation results further suggest that strengthening psychological capacities alone may be insufficient when students perceive high levels of employment uncertainty. Institutional career services may improve effectiveness by providing clearer labor market information, realistic career guidance, and structured transition support that reduces uncertainty perceptions and helps students translate psychological strengths into confident employability evaluations. At a broader level, the findings underscore the importance of aligning educational support with labor market transparency and transition assistance. Policies that expand access to employment information, internship pathways, and early career support mechanisms may reinforce the connection between students’ internal psychological resources and successful adaptation to employment environments. Taken together, the results highlight that employability development is most effective when attention is directed simultaneously toward individual psychological capacities and the broader labor market context in which graduates begin their careers.

## Data Availability

The raw data supporting the conclusions of this article will be made available by the authors, without undue reservation.
